# AQM based on the queue length: A real-network study

**DOI:** 10.1371/journal.pone.0263407

**Published:** 2022-02-01

**Authors:** Marek Barczyk, Andrzej Chydzinski

**Affiliations:** Department of Computer Networks and Systems, Silesian University of Technology, Gliwice, Poland; Hankuk University of Foreign Studies, KOREA, REPUBLIC OF

## Abstract

Active Queue Management (AQM) is recommended by Internet Engineering Task Force to mitigate the bufferbloat phenomenon in the Internet. In this paper, we show the results of comprehensive measurements carried out in our university network, in which a device with an AQM algorithm, designed and programmed for this purpose, was running. The implemented AQM algorithm was based on the dropping function, i.e. arriving packets were dropped randomly, with the probability being a function of the queue length. Several different dropping function forms, proposed in the networking literature, were used, in addition to the classic FIFO queue (no AQM). The experiment lasted over a month, during which the state of the network was measured and recorded several thousand times. This made the results independent of the natural fluctuations of the users’ behavior and the network load. Conclusions on the general performance improvement offered by the implemented AQM, as well as the differences in the performance between particular forms of the dropping function, were reached. Some of these conclusions differ from those drawn previously from simulations. This underlines the need for carrying measurements of new AQMs in real, operating networks, with complex, natural traffic.

## 1 Introduction

In the early Internet, the buffers at network devices were meant to mitigate the losses caused by the statistical multiplexing of flows incoming from different directions. Namely, the random nature of incoming flows caused occasional bursts of traffic at nodes. These bursts had to be stored in buffers, before being forwarded. This was initially the only purpose of buffers. At this early stage, the congestion control in the Internet was poor, what led to a few spectacular congestion collapses and forced the search for a new, efficient method of congestion control. Such method, the Reno algorithm, was found and widely implemented, what eliminated congestion collapses and improved the inter-flow fairness.

Unfortunately, the new algorithm, and its descendants used today, exploit the buffers in a different way. They constantly increase the sending rates of individual end systems, until one or more buffers on the transmission path get full, causing either long queueing delays, or packet loss, or both. In this way, by noticing the long delays and/or losses, modern congestion control mechanisms are informed that the maximum possible sending rate has been achieved.

As we can see, there is a clash between the original purpose of buffers, and their usage by the congestion control mechanism, which tends to fill the buffers, no matter how big they are. This, of course, has some negative consequences. The most profound and widely described is called *bufferbloat*, [1, 2]. The presence of bufferbloat means simply that a substantial fraction of buffers in the Internet contains long queues of packets, much longer than necessary for proper functioning of the network. These long queues cause long queueing delays and, in general, longer than necessary packet delivery times.

The cure for bufferbloat is well known and called Active Queue Management (AQM). It exploits the idea that some arriving packets should be dropped before the queue in a buffer gets long, informing in this way end systems about the need to reduce their sending rates. AQM has been recommended, for instance, by Internet Engineering Task Force in the RFC 7567 document, [3].

Based on theoretical and simulation studies, many AQM algorithms have been proposed so far. In some algorithms, a deterministic decision on each arriving packet is reached, whether to drop it or not, [4, 5]. In the majority of propositions, however, each packet can be dropped randomly, with a probability being constantly re-calculated with regard to the current and recent network conditions, e.g. [6–11]. Sometimes, the methods of re-calculation of the dropping probability are very refined and exploit the fuzzy logic [12, 13], genetic algorithms [14] or neural networks [15, 16].

In particular, in [12], an autonomic, intelligent and stateless proactive fuzzy logic AQM, called PFL, is proposed. PFL uses an efficient non-linear fuzzy control to detect the network congestion and tune the dropping probability to it. Moreover, the built-in self configuration mechanism, based on the steady-state packet loss ratio, enables it to control the PFL output dynamically. In [13], a method called FPID is proposed, in which the RED algorithm works with the fuzzy proportional integral derivative approach. A very good performance is achieved, due to the application of the fuzzy theory, combined with the classic PID theory. In [14], a linear-quadratic-optimal controller is designed, based on the linear control theory for an AQM router. One of the crucial and difficult steps of the algorithm, i.e. finding the weight matrices, is based on the genetic algorithm, what constitutes an alternative approach for the time-varying feedback loop control. In [15], the performance of four algorithms based on neural networks, i.e. PID, AN-AQM, FAPIDNN and NRL, is studied. All the four algorithms exhibit a good accuracy and small queue length jitter in simulations. Their disadvantage is, however, the lack of a formal proof of stability. Finally, in [16], a yet another AQM idea based on neural networks is introduced. Namely, a combination of neural networks, distributed adaptive control and barrier Lyapunov function is used to guarantee that the tracking errors approach a pre-defined zone, independently of initial conditions.

Now, a very important class of AQM algorithms consists of those, in which the probability of dropping an incoming packet is a function of the queue size. This function is called the dropping function. In the first algorithm of this type, [17], a simple linear dropping function was proposed. Then some other shapes of the dropping function were investigated, among them a broken line with increasingly steeper slope [18], en exponential function [19], polynomials of second and third degree, [20, 21], a mixture of linear and cubic functions, [22], and a product of a linear function with its logarithm, [23].

An AQM based on the dropping function may not be sometimes as good as best solutions exploiting advanced machine-learning methods, mentioned above. It is, however, extremely simple conceptually, easy in implementation, and, as we will see herein, offers a substantial improvement of the performance, when compared with no AQM at all. Moreover, the stability of AQM based on the dropping function has been formally proven, [24].

So far, AQM algorithms based on the dropping function have been widely studied via mathematical modeling and computer simulation. We are, however, unaware of any published results of tests of these algorithms implemented in a real device, and carried out in a real, fully operational network.

In this paper, we show results of measurements of a fully operational network of our university campus, in which a device with AQM based on the dropping function, designed and programmed by us, was running. Nine different dropping function forms, proposed in the literature, have been tested and compared with each other, and with the classic FIFO-tail-drop queue (no AQM). The experiment lasted over a month, during which the state of the network was measured and recorded several thousand times, with random changes of the dropping function. This made the results independent of the natural fluctuations of the users behavior and the network load. Each measurement lasted as long as 2 million packets passed through the AQM device. During such period, several performance characteristics were recorded, including the network load, the average queue size and its standard deviation, the packet loss ratio and the packet burst ratio. These detailed characteristics were then aggregated using cost functions (6) and (7), defined in the next section, which enabled us to assess the overall impact of particular dropping functions, and the lack of AQM.

The device with AQM, used in the measurements, was an x86, multi-core server, running Linux operating system, with implemented dropping function mechanism. DPDK cards and software, [25], were used to provide fast packet processing. They made it possible to process and forward the arriving traffic with the full speed of the output interface, i.e. 1Gb/s. Prior to the measurements carried out in a real network, our AQM device has been tested in a networking laboratory, using artificial traffic from Spirent generator. The results of these tests were described in [26]. It is important to note, however, that tests in a local lab cannot properly expose advantages or disadvantages of an AQM scheme, for several reasons. The most important reason is that the round trip propagation times (RTPTs) are unrealistically small in a local lab (below 1ms). In the measurements described in this paper, many flows traversing the AQM device experienced much longer RTPTs, of tens or hundreds milliseconds. Such RTPTs are hard to mimic in a local lab. On the other hand, it is well-known that high RTPTs have a profound impact on the behavior of the TCP congestion control mechanism, and, as a consequence, the AQM algorithm (e.g. [27]). Therefore, results of AQM tests obtained in a local lab may differ from those obtained in a real, working network.

Finally, it is worth mentioning that queueing models with the dropping function have been studied recently using the mathematical methods of classic queueing theory, with different assumptions on the arrival stream and the service time distribution, e.g. [24, 28–30]. Some of these theoretical results have been used to parameterize the dropping functions, implemented in the AQM device to provide a specific performance goal, i.e. the average queue size of 50 packets at the network load of 1.

The rest of the paper is organized in the following way. In Section 2, the mathematical results used to parameterize the dropping functions in the AQM device are recalled first. Then, nine formulas for dropping functions implemented in the AQM device are given, as well as the definitions of the performance characteristics collected in the experiment. The academic network, in which the measurements were carried out, is described in Section 3. In Section 4, the design of the device with the AQM algorithm based on the dropping function is presented. Namely, the internal structure exploiting the multi-core architecture, the actual packet flow and processing, as well as the advantages of using the DPDK framework, are described. Then, in Section 5, the results of the measurements are presented and discussed. In particular, five graphs depicting the mean queue length, its standard deviation, the loss ratio, the burst ratio and the total impairment factor, for different loads and dropping functions, are presented and discussed. In addition, a table with all the performance characteristics, averaged over short load intervals, are shown and discussed. The final conclusions and recommendations are given in Section 6.

## 2 Theoretical results, dropping functions and characteristics used in the experiment

In what follows, the dropping function will be denoted by *d*(*n*), where *n* is the queue length upon a packet arrival, and *d*(*n*) is the probability of dropping this packet. Naturally, for every *n* we have 0 ≤ *d*(*n*) ≤ 1.

The particular shapes of the dropping function, used in the measurements, were taken from the literature (there will be references next to formulas). The specific parameterization of each of them has been carried out using a model of the queue with the dropping function taken from [24]. Namely, the following theorem was proven there.

**Theorem 1**. *If the interarrival time distribution is exponential with parameter* λ *and the service time distribution is exponential with parameter μ*, *then the probability P*_*k*_ *that the queue size is k in the steady state is equal to*:

$$\begin{array}{c} P_{0}=\frac{1}{1+\sum_{k=1}^{\infty }\rho^{k}\prod_{i=0}^{k-1}[1-d(i)]}, \end{array}$$ (1)

$$\begin{array}{c} P_{k}=\rho^{k}P_{0}\prod_{i=1}^{k}[1-d(i-1)],\;\;\;\;k=1,2\ldots , \end{array}$$ (2)

*the average queue size and its standard deviation are equal to*:

$$\begin{array}{c} EX=\sum_{k=0}^{\infty }kP_{k},\;\;\;\;DX=\sqrt{\sum_{k=0}^{\infty }k^{2}P_{k}-{\left(EX\right)}^{2}}, \end{array}$$ (3)

*respectively, the loss ratio is equal to*:

$$\begin{array}{c} L=1-\frac{1-P_{0}}{\rho }, \end{array}$$ (4)

*while the average response time of an accepted packet is equal to*:

$$\begin{array}{c} ER_{a}=\frac{EX\rho [1+\sum_{k=1}^{\infty }\rho^{k}\prod_{i=0}^{k-1}\left(1-d\left(i\right)\right)]}{\lambda \sum_{k=1}^{\infty }\rho^{k}\prod_{i=0}^{k-1}(1-d(i))}, \end{array}$$ (5)

*where ρ* = λ/*μ is the load offered to the queue*.

**Fig 1:**
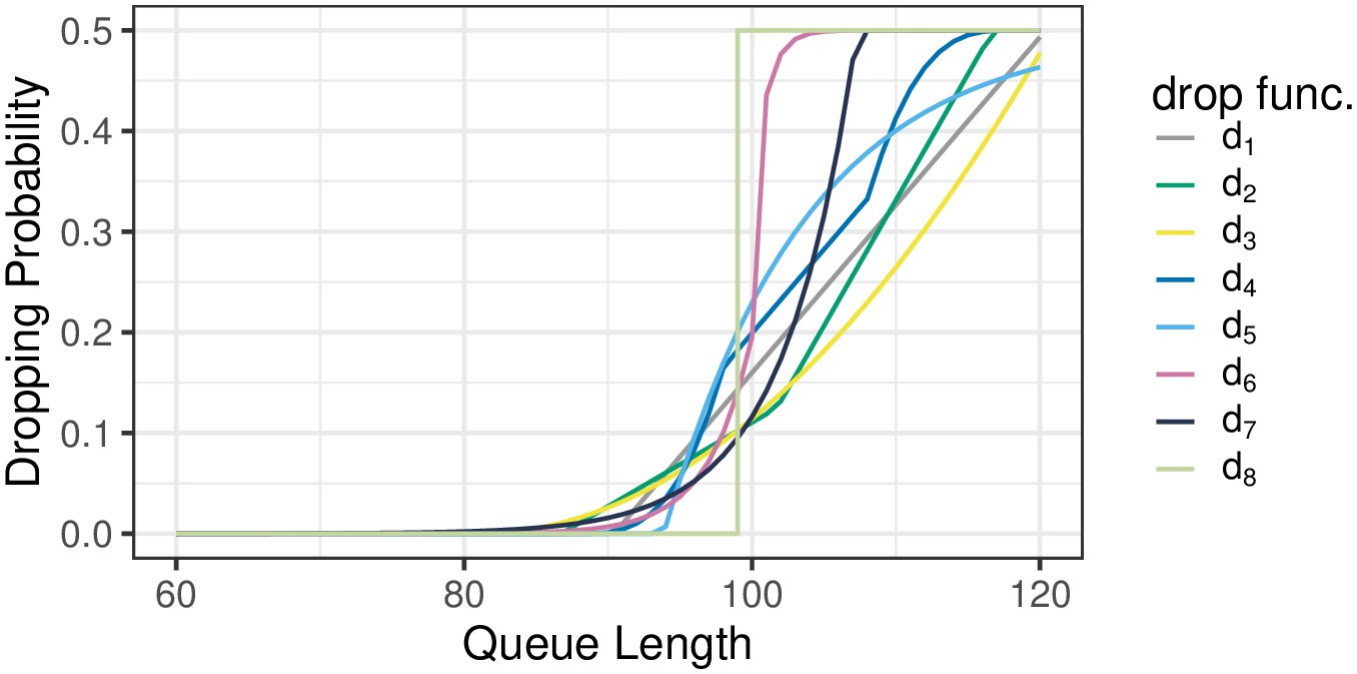
Dropping functions *d*_1_-*d*_8_.

Using this theorem, nine different dropping function types were parameterized in such a way, that each of them provides EX=50, i.e. the average queue size of 50 packets, for *ρ* = 1, (see [26]). The resulting dropping functions have the following forms (depicted also in Fig 1):

- small buffer shape, see e.g. [31]:
  $$\begin{array}{c} d_{0}\left(n\right)=\{\begin{array}{ccc} \;0, & \;\text{if}\; & n<100,\\ \;1, & \;\text{if}\; & n\geq 100, \end{array} \end{array}$$
- RED shape, see e.g. [17]:
  $$\begin{array}{c} d_{1}\left(n\right)=\{\begin{array}{ccc} \;0, & \;\text{if}\; & n<91,\\ \;0.016666n\;-\;1.506666, & \;\text{if}\; & 91\leq n<121,\\ \;0.5, & \;\text{if}\; & n\geq 121, \end{array} \end{array}$$
- GRED shape, see e.g. [18, 28]:
  $$\begin{array}{c} d_{2}\left(n\right)=\{\begin{array}{ccc} \;0, & \;\text{if}\; & n<87,\\ \;0.008333n\;-\;0.722833, & \;\text{if}\; & 87\leq n<102,\\ \;0.025n-2.4185, & \;\text{if}\; & 102\leq \;n<\;117,\\ \;0.5, & \;\text{if}\; & n\geq 117, \end{array} \end{array}$$
- NLRED shape, see e.g. [20]:
  $$\begin{array}{c} d_{3}\left(n\right)\;=\;\{\begin{array}{ccc} \;0, & \;\text{if}\; & n<81,\\ \;0.000312{\left(n-80.88\right)}^{2}, & \;\text{if}\; & 81\leq n<\;121,\\ \;0.5, & \;\text{if}\; & n\geq 121, \end{array} \end{array}$$
- TRED shape, see e.g. [22]:
  $$\begin{array}{c} d_{4}\left(n\right)=\{\begin{array}{ccc} \;0, & \;\text{if}\; & n<89,\\ \;4.5{\left(\frac{n-88.05}{30}\right)}^{3}, & \;\text{if}\; & 89\leq n<99,\\ \;0.016666n\;-\;1.467499, & \;\text{if}\; & 99\leq \;n<\;109,\\ \;4.5{\left(\frac{n-118.05}{30}\right)}^{3}+0.5, & \;\text{if} & 109\;\leq \;n<\;119,\\ \;0.5, & \;\text{if}\; & n\geq 119, \end{array} \end{array}$$
- REM shape, see e.g. [19]:
  $$\begin{array}{c} d_{5}\left(n\right)=\{\begin{array}{ccc} \;0, & \;\text{if}\; & n<94,\\ \;0.5-0.5e^{-(n-93.86)/10}, & \;\text{if}\; & n\geq 94. \end{array} \end{array}$$
- S shape, see e.g. [24]:
  $$\begin{array}{c} d_{6}\left(n\right)=\{\begin{array}{ccc} \;0.25e^{\frac{n-100.74}{3}}, & \;\text{if}\; & n<101,\\ \;0.5-0.25e^{\frac{100.74-n}{3}}, & \;\text{if}\; & n\geq 101, \end{array} \end{array}$$
- exponential shape, see e.g. [24]:
  $$\begin{array}{c} d_{7}\left(n\right)=\{\begin{array}{ccc} \;0.5e^{\frac{n-107.3}{5}}, & \;\text{if}\; & n<108,\\ \;0.5, & \;\text{if}\; & n\geq 108, \end{array} \end{array}$$
- constant shape:
  $$\begin{array}{c} d_{8}\left(n\right)=\{\begin{array}{ccc} \;0, & \;\text{if}\; & n<99,\\ \;0.5, & \;\text{if}\; & n\geq 99. \end{array} \end{array}$$

Besides the nine functions listed above, the classic FIFO algorithm (no dropping function) was used. In such case, the default buffer size was 300 packets.

Therefore, we used 10 queueing schemes in total—nine with the dropping functions and one without. The AQM device switched randomly between the ten, so that there was no correlation between the particular day or time of the day and the queueing scheme used. The switching scheme will be described in detail in Section 5.

Each measurement lasted as long as 2 million packets passed through the AQM device. During such period, several performance characteristics were recorded, in particular: the load of the AQM device, *ρ*, the average size of arriving packet and its standard deviation, the average queue length in packets (EX) and its standard deviation (DX), the loss ratio (*L*), and the burst ratio (*B*).

All these characteristics are rather well known and commonly used. Nevertheless, to avoid any misunderstandings, we will clarify now how we understood and measured them. The load was understood as the average arrival rate of packets bound to the output link, divided by the capacity of this link, i.e. 1Gb/s. The loss ratio was measured as the fraction of packets which were dropped during the measurement, either by the dropping function mechanism, or by the overflowed buffer. Finally, the burst ratio was understood as the ratio of the average number of packets dropped in a row, divided by the theoretical number of packets dropped in a row, that should have been observed for a purely random and independent loss, [32]. By this definition, *B* informs us about the statistical structure of packet losses, i.e. their tendency to cluster together. If *B* = 1, then the losses seem to occur randomly and independently of each other. If *B* < 1, then the losses tend to occur separately, as single events. If *B* > 1, then the losses tend to group together, in long series. The latter is known to influence negatively important networking services, e.g. real time multimedia transmissions. (It is also worth mentioning, that *B* is not the only parameter used to characterize the structure of losses; see e.g. [33, 34] for other approaches).

Due to the fact that high values of both loss ratio and burst ratio have negative (but different) influence on the quality of transmission, it is useful to estimate their combined negative effect using one factor. Such an impairment factor, combining *L* and *B*, have been proposed in [35], and has the following form:

$$\begin{array}{c} I=I_{e}+\left(95-I_{e}\right)\frac{100L}{100L/B+R}, \end{array}$$ (6)

with *I*_*e*_ and *R* denoting some constants (by default, *I*_*e*_ = 0 and *R* = 4.3). Therefore, in our experiment, after each measurement of *L* and *B*, the factor *I* was computed, to allow for an easy assessment of the total deterioration of transmission caused by losses, including their structure.

Obviously, the most important parameter, exposing the occurrence of the bufferbloat, is the average queue size. Of almost the same importance is the factor *I*. To assess their combined negative impact, we can use the following global impairment factor:

$$\begin{array}{c} C=EX+I. \end{array}$$ (7)

Finally, the standard deviation of the queue size can be considered as a supplementary characteristic. It reflects the stability of the queue size, provided by the AQM mechanism and influences the delay jitter of the transmission.

## 3 Academic network used in the experiment

**Fig 2:**
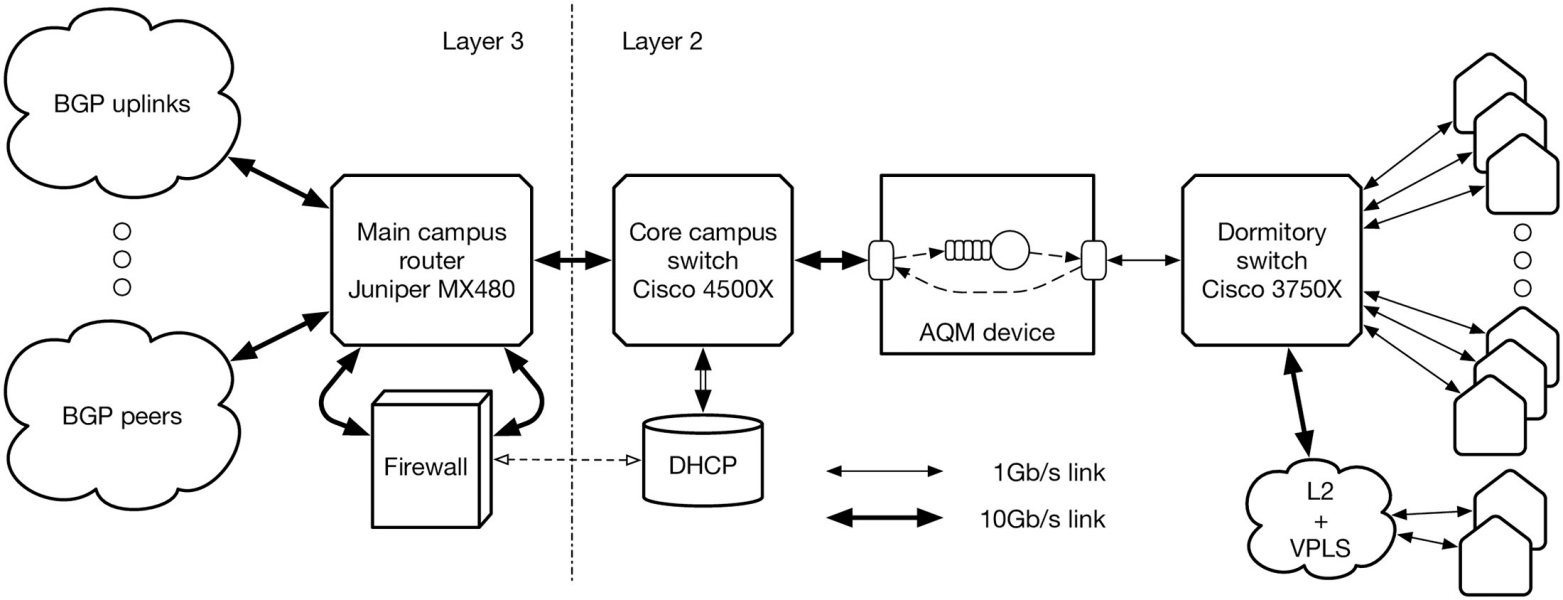
University network used in the experiment.

The scheme of the campus network of the Silesian University of Technology, used in the measurements, is shown in Fig 2. The network routing is handled by Juniper MX480 core router (on the left of Fig 2). During the experiment, the AQM device with the dropping function (in the middle) was connected into the network part servicing student dormitories (on the right). Each dormitory building has dedicated 802.1q virtual LAN. Two of the buildings are located in neighboring cities, thus to reach them, the VLANs follow an MPLS cloud with VPLS.

The network in each dormitory is an autonomous network, administered by a different entity. From our experiment point of view, each such network is a different sub-ISP network and it connects users using layer 2 devices. The access to the Internet, DHCP and security are handled by the devices in the campus network. Each end system in a dormitory has a public IP address, so there is no NAT on the path to the Internet.

During the measurements, the aggregated traffic from the Internet towards the dormitories was typically up to 1.2Gb/s, rarely exceeding this value. The return traffic was about 10 times smaller and it was unaffected by the AQM device. The TCP/UDP proportions varied from 75/25 to 50/50 percent. The average packet size was about 1300 bytes, with the standard deviation of about 450bytes.

The network measurements have been formally approved by the Vice Rector for Science and Development of the Silesian University of Technology, prof. Marek Pawełczyk.

## 4 AQM device used in the experiment

The AQM device, shown in the middle of Fig 2, was an x86-multi-core server running Linux operating system. This device was designed and programmed by us to test the dropping function mechanism in real networks. What is important, DPDK cards and software were used in this device, what allowed it to achieve the processing and forwarding speed exceeding by far the bit rates actually occurring in the experiment. First of all, the DPDK framework allows disabling the interrupts on the participating network interfaces. To further improve the performance, CPU cores can be assigned to separate tasks performed concurrently. Further speedup is gained by processing buffer descriptors, pointing to memory locations with packets, rather than actual packets.

**Fig 3:**
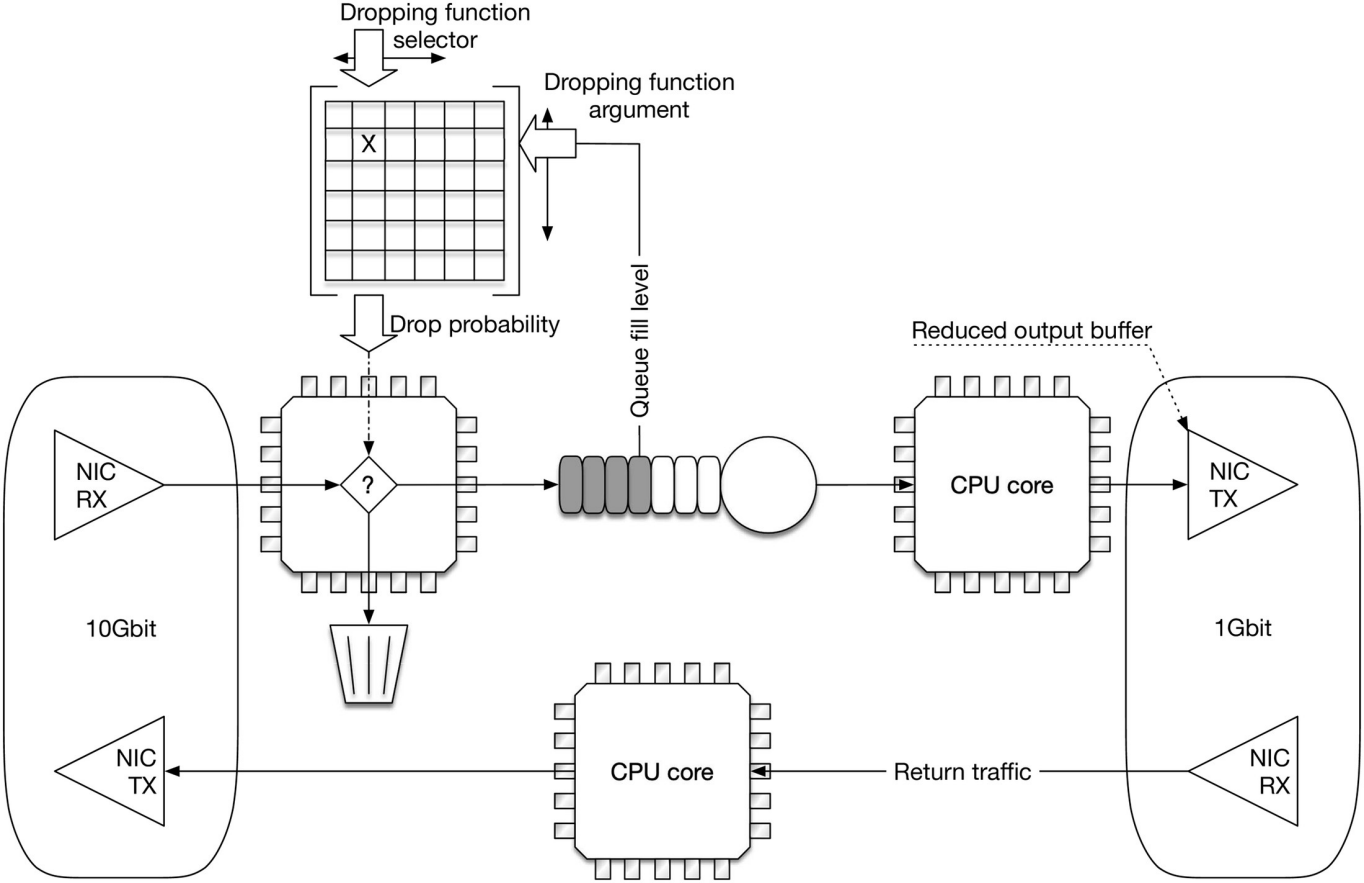
Scheme of the AQM device with the selectable dropping function.

The schematic internal structure of our AQM device is depicted in Fig 3. The interface on the right has the speed of 1Gb/s, which constitutes the potential bottleneck for the aggregated traffic bound to dormitories. The interface on the left runs at the speed of 10Gb/s, which enables it to receive traffic exceeding 1Gb/s.

In general, each CPU core in Fig 3 runs in an infinite loop, polling one buffer for packets, performing perhaps some tasks, and sending the packets to some other buffer.

In particular, the CPU core on the left of Fig 3 performs the actual dropping of packets, depending on the selected dropping function and the current queue size. Unfortunately, the DPDK framework does not allow querying the NIC queue size, due to the lengthy PCIe operation required to access NIC processor registers. Therefore, to be able to read the queue size, we had to introduce an intermediate queue in the memory (in the middle of Fig 3). It exploits the ring-buffers library, built into DPDK, and enables the concurrent atomic access, which does not require locking of the queue for reading or writing. Thus the current size of this queue can be easily calculated. The CPU core on the left does so after reading a packet from the left NIC RX queue. Then it queries the dropping probability from a tabularized dropping function, calculates a random number, and combines that information into a drop or no-drop decision. If the packet is dropped, then its memory is freed. Otherwise, the packet is enqueued into the intermediate queue. From this queue, the CPU core on the right reads packets and outputs them to the right NIC TX buffer. This buffer has a small size of 4 packets, to minimize its influence on the performance of the device.

The CPU core in the middle of Fig 3 simply forwards packets on the uncongested return path.

The secondary task of the AQM device is to carry out the measurements during the course of the experiment. This task is handled also by the CPU core on the left. All the characteristics are gathered in every cycle performed by this core. As it keeps the current queue size all the time (an argument of the dropping function), the average queue size and its standard deviation can be computed easily. The same core is used to drop packets, so the loss ratio and burst ratio can be obtained with equal ease.

To be sure that all these operations do not cause any performance overhead influencing the measurements, we tested the device using a hardware generator. Namely, a high load of 3Gb/s of small packets (100-bytes long) was generated. No performance issues were observed under such load. Both the bit rate and the number of packets processed per second in this stress test exceeded by far what was then met in the real experiment. In this way, we made sure that the actual measurements were not affected by any issues with the performance of the device.

## 5 Experiment results and discussions

The experiment lasted continuously over a month. On the hour, i.e. at 7:00, 8:00, 9:00, etc, the queueing algorithm was switched randomly between *d*_0_-*d*_8_ and no AQM, with the period of operation of one hour. Namely, an integer random number from set {0, 1, …, 9} was generated first. If number 9 was generated, the AQM device operated with no AQM for the next hour. If number *i* ∈ {0, 1, …, 8} was generated, the device used *d*_*i*_ dropping function for the next hour. An hour later, a new integer was generated, the queueing algorithm switched to a different dropping function, and so on.

This operation scheme was meant to assure that every dropping function worked in every networking conditions, i.e. in the morning, in the middle of the day, at night, at the beginning of the week, at the end of the week, during the weekend, when the load was high, low, moderate, etc. The one hour operation time of a particular dropping function was chosen as being long enough to allow the algorithm to reach its steady-state operation regime and short enough to provide approximately stable networking conditions, i.e. the level of users activity and the network load.

During the operation of a particular dropping function, measurements of the performance characteristics provided by this function were carried out. In every measurement, the current dropping function, the load, the average queue size and its standard deviation, the packet loss ratio and the packet burst ratio were collected. In addition, the cost functions values (6) and (7) were computed. Each measurement lasted as long as 2 millions packets passed trough the AQM device.

**Fig 4:**
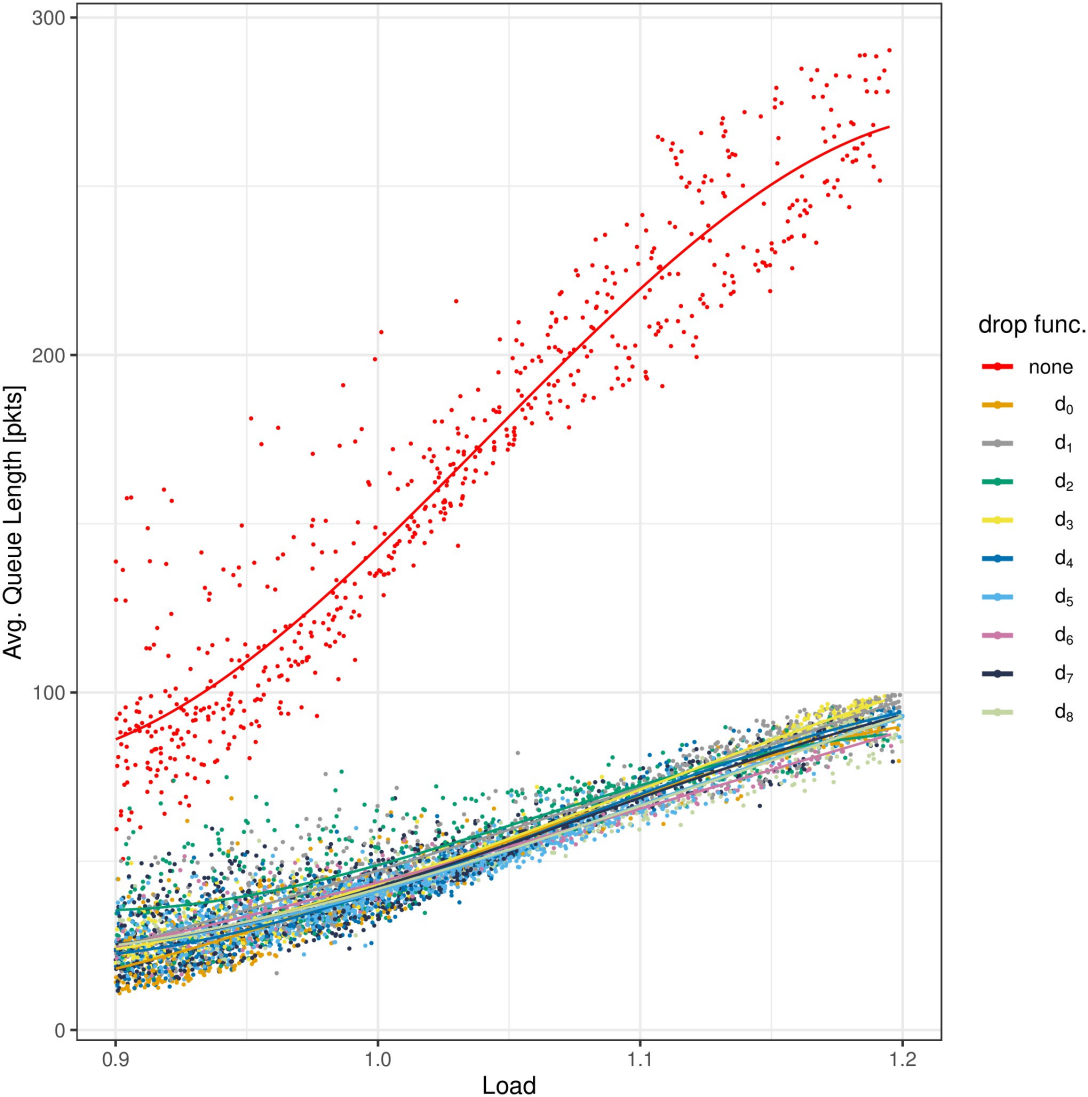
The average queue length, EX, versus load.

**Fig 5:**
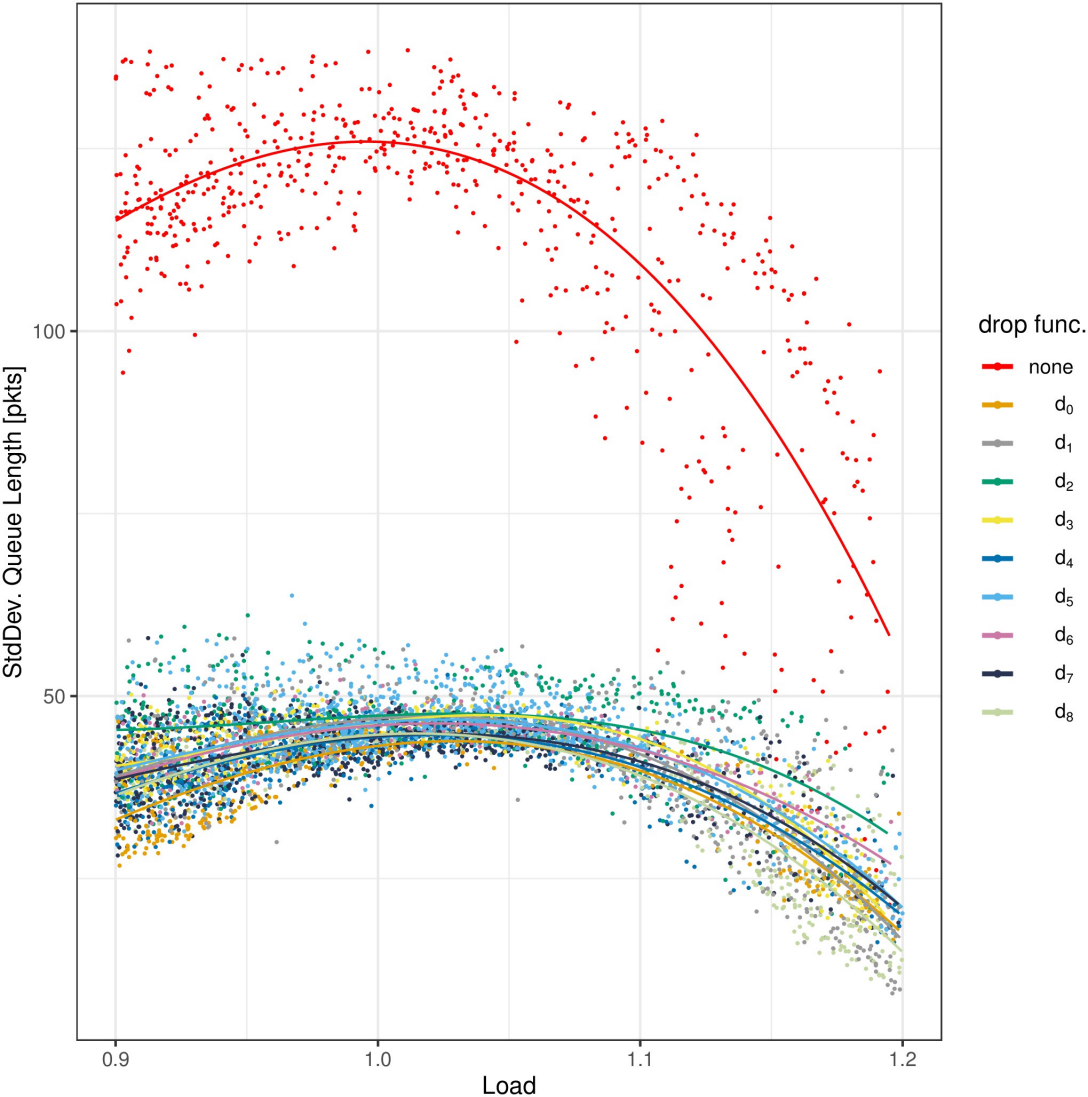
The standard deviation of the queue length, DX, versus load.

**Fig 6:**
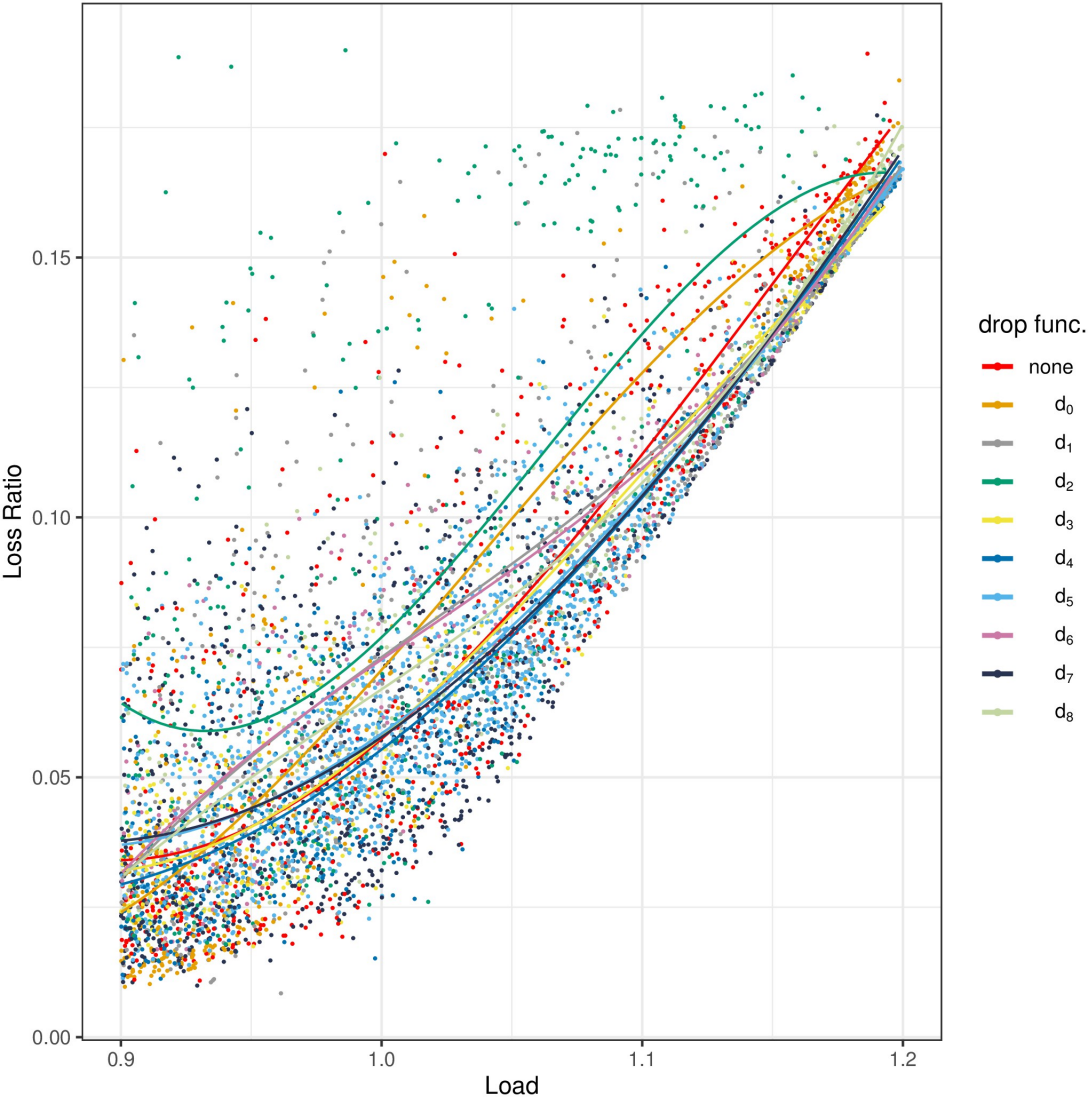
The loss ratio, *L*, versus load.

**Fig 7:**
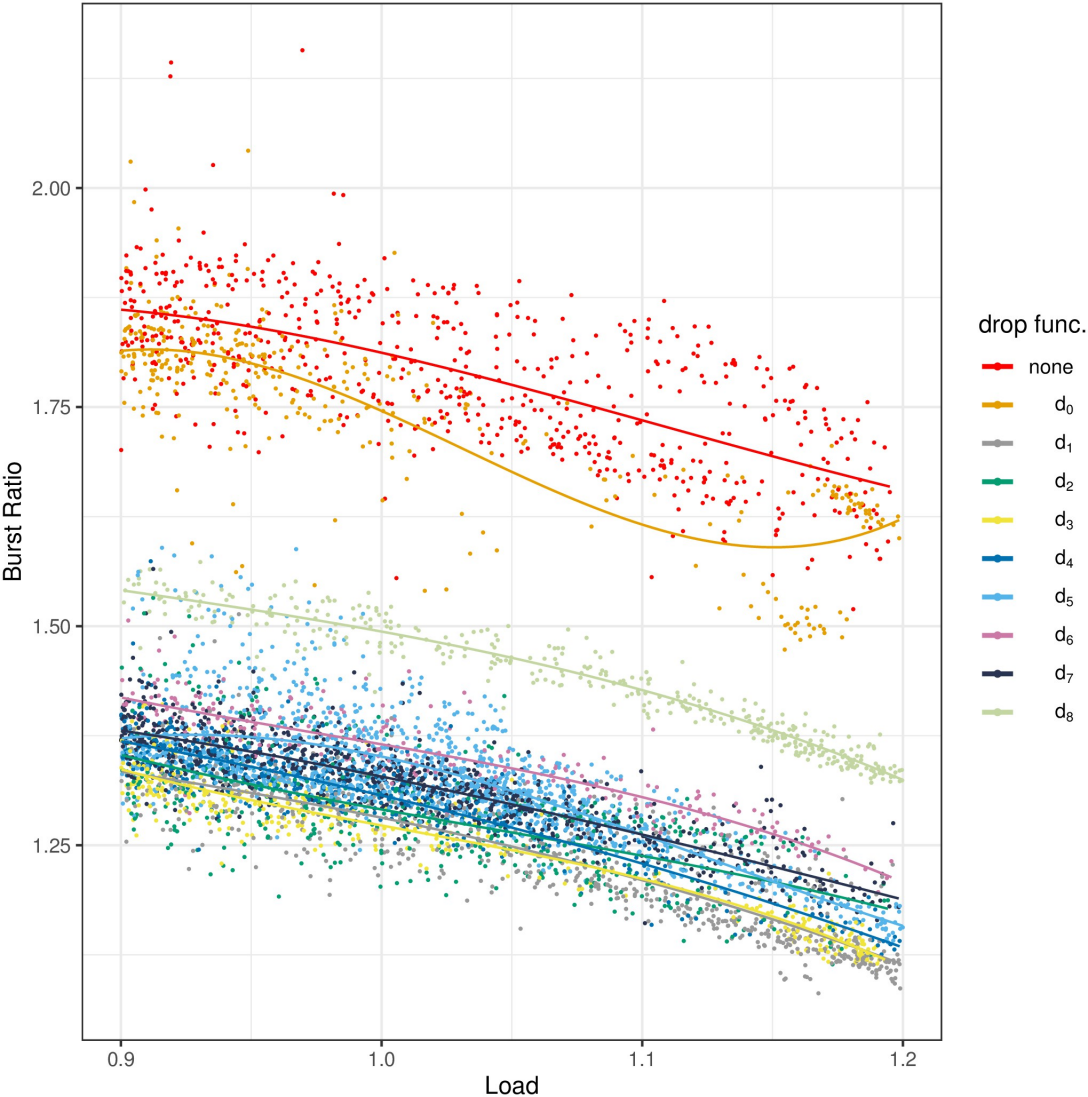
The burst ratio, *B*, versus load.

**Fig 8:**
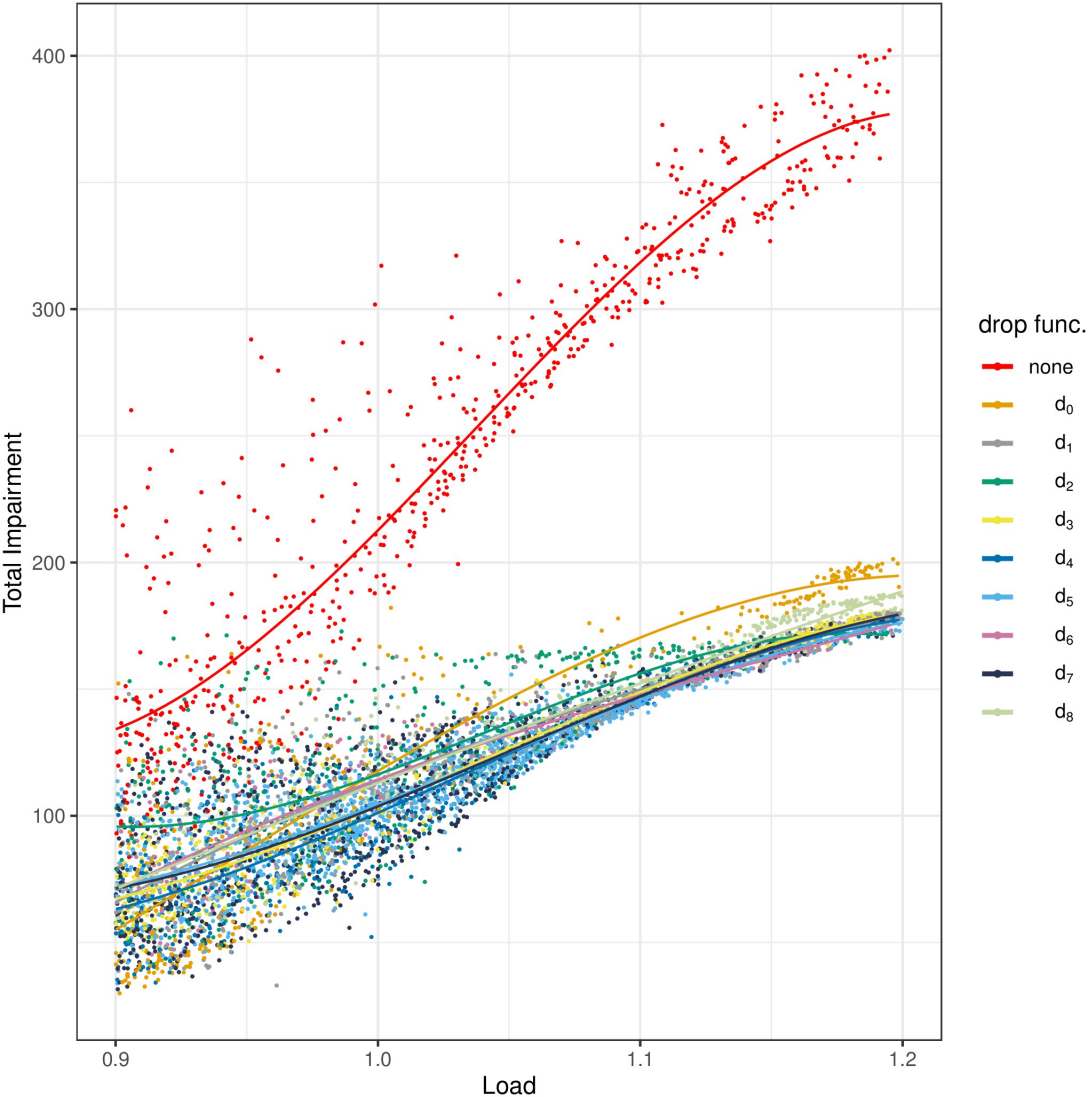
The total impairment factor, *C*, versus load.

The results of the measurements are depicted in Figs 4–8. All figures have the load on the horizontal axis, and the characteristic of interest on the vertical axis. In particular, in Fig 4 we see the average queue size, in Fig 5—the standard deviation of the queue size, in Fig 6—the packet loss ratio, in Fig 7—the packet burst ratio, while in Fig 8—the total impairment factor, *C*.

In these graphs, each individual measurement is presented as a single, colored dot, where the color represents the particular dropping function, the same as in Fig 1. As we can see, for one value of the load and one dropping function, we can have several, quite different measurements of the performance characteristic. This is natural in a real networking environment. It can be caused, for instance, by different proportions of the TCP and UDP traffic, at different times. Therefore, a third-degree polynomial is fitted, using the generalized additive model, to all measurements within a particular dropping function. These polynomials are plotted as the curves of the same color, as dots obtained for a particular dropping function.

All the graphs begin with the load of 0.9. There were also measurements with the load less than 0.9, but such results are less interesting, as the queue sizes and losses are small for a low load, no matter if AQM is used, or not. Finally, there were very few measurements for *ρ* > 1.2, therefore the graphs end at the load of 1.2.

Now, as we can see in Fig 4, all dropping functions *d*_0_-*d*_8_ provide similar queue sizes within the whole range of *ρ*. The lack of the dropping function, on the other hand, results in a significantly (several times) longer queues, i.e. the bufferbloat. Therefore, when the bare queueing delay is taken into account, the application of the dropping function is clearly advantageous, no matter which shape of the nine is used. Note also that all the dropping functions induce the average queue size pretty close to 50 packets for *ρ* = 1, which was the design goal while parameterizing these functions by means of the mathematical model of [24].

In Fig 5 we can observe the stability of the queue size. All the dropping functions provide a similar, good stability, with slightly worse results for *d*_2_. Again, the lack of the dropping function results in a much worse deviation of the queue size.

All of the observations made so far are consistent with the previous studies conducted via simulations (see e.g. [36]).

However, Fig 6 is quite surprising and inconsistent with the results of the previous studies. As indicated by simulations, an application of the dropping function should improve the queueing delay, but always at the cost of an increased loss ratio. This is not confirmed in Fig 6, which is a good news. On the contrary, most of the used queueing schemes, including no AQM, induce similar loss ratios. What is more, the smallest loss ratios are not obtained when no AQM is used, but for one of the dropping functions. Namely, *d*_4_ provides the lowest loss ratios in the whole range of the offered load. On the other hand, a little worse than average results are obtained for *d*_2_ and *d*_0_.

In Fig 7, the burst ratio can be studied. As we can see, three queueing algorithms induce visibly worse results. In particular, especially bad results are obtained for no AQM and for the trivial dropping function, *d*_0_ (small buffer). They are both far worse than the results for all non-trivial dropping functions, *d*_1_-*d*_7_, which are rather close to each other. Quite bad results are also obtained for another trivial dropping function, *d*_8_. Furthermore, the absolute values of *B* obtained in the measurements for no AQM case are very high. In the whole range of the load, they oscillate around 1.75, sometimes even exceeding 2. Therefore, it is really important to decrease such high values of *B*, to values much closer to 1, which can be easily achieved by an application of a proper dropping function.

Finally, in Fig 8, the total impairment factor, *C*, is depicted. It combines the impact of the queueing delay with the loss impairment, given in formula (7). As we can see, the no-AQM case is far worse than anything else. Among the dropping functions, the trivial function *d*_0_ performs visibly worse than others. This is consistent with the results of the previous studies.

We may also notice in Fig 8, that in general *d*_4_ is better, while *d*_2_ is worse, than any other non-trivial dropping function, *d*_1_-*d*_7_. This is inconsistent with the results of the previous studies—in simulations, the convex dropping function *d*_2_ prevailed. It must be stressed, however, that the differences between *d*_4_ are *d*_2_ small, if compared with no AQM at all.

Summarizing these considerations, we can draw the following general picture. All the non-trivial dropping functions, *d*_1_-*d*_7_, provide a significant improvement of the performance, if compared with no AQM, when both the queuing delay and losses are taken into account. The trivial dropping functions, *d*_0_ and *d*_8_, provide a similar improvement of the queueing delay, but their performance is inferior in terms of the structure of losses.

There are some differences in the performance among the proper dropping functions, *d*_1_-*d*_7_. For instance, *d*_4_ provides the best loss ratios, *d*_3_—the best burst ratios, etc. However, these differences are not crucial—they are far lower than the difference between an application of a proper dropping function, and no AQM at all.

The same conclusions, that were drawn from Figs 4–8, can also be drawn from Table 1. In this table, the average measurements obtained for different load ranges are presented. Namely, the following load ranges, are used: (0.90–0.94], (0.94–0.98], …, (1.14–1.18]. Within each load range, we have the average value of a particular characteristic, e.g. the queue size, for every dropping function separately. Moreover, the worst result within a particular load range and particular characteristic is boxed. On the other hand, the best result within a particular load range and particular characteristic is printed in a bold font.

**Table 1:** Average performance characteristics in different ranges of the network load.

Load, *ρ*	Dropping function	Avg. queue, EX	StdDev. queue size	Loss ratio, *L*	Burst ratio, *B*	Total impairment, *C*
(0.9–0.94]	none	93.20	120.71	0.0347	1.872	146.80
	*d*_0_	**21.19**	**36.61**	**0.0292**	1.839	**68.33**
	*d*_1_	30.58	44.07	0.0450	1.346	86.49
	*d*_2_	36.90	47.86	0.0601	1.370	102.62
	*d*_3_	26.66	42.01	0.0344	**1.328**	74.06
	*d*_4_	24.77	39.78	0.0325	1.365	71.02
	*d*_5_	27.27	42.50	0.0389	1.390	79.35
	*d*_6_	27.94	41.57	0.0408	1.413	81.83
	*d*_7_	27.21	41.39	0.0388	1.391	79.22
	*d*_8_	26.49	39.69	0.0369	1.538	78.81
(0.94–0.98]	none	113.12	125.85	0.0441	1.858	175.91
	*d*_0_	**31.23**	**41.81**	0.0485	1.791	96.97
	*d*_1_	37.75	46.33	0.0572	1.330	100.95
	*d*_2_	40.41	48.35	0.0638	1.358	107.74
	*d*_3_	34.05	44.94	0.0446	**1.306**	89.00
	*d*_4_	32.22	42.80	**0.0427**	1.340	**86.42**
	*d*_5_	33.47	45.15	0.0471	1.380	91.51
	*d*_6_	35.75	44.34	0.0569	1.390	100.18
	*d*_7_	34.87	43.99	0.0494	1.368	94.21
	*d*_8_	36.11	43.40	0.0573	1.518	103.53
(0.98–1.02]	none	143.22	127.44	0.0562	1.828	215.63
	*d*_0_	41.78	**44.32**	0.0707	1.782	123.03
	*d*_1_	47.07	47.81	0.0730	1.302	117.06
	*d*_2_	48.44	48.36	0.0749	1.315	119.63
	*d*_3_	41.99	46.27	0.0536	**1.279**	101.99
	*d*_4_	**39.94**	44.45	**0.0530**	1.317	**100.44**
	*d*_5_	40.68	46.43	0.0580	1.353	104.85
	*d*_6_	44.02	46.80	0.0742	1.370	116.57
	*d*_7_	41.36	44.80	0.0556	1.344	103.99
	*d*_8_	42.03	44.55	0.0676	1.501	114.96
(1.02–1.06]	none	174.02	125.95	0.0773	1.803	259.56
	*d*_0_	54.12	**44.14**	0.0928	1.699	144.43
	*d*_1_	56.80	47.40	0.0883	1.270	131.34
	*d*_2_	58.32	48.12	0.0950	1.295	135.88
	*d*_3_	53.47	47.63	0.0769	**1.262**	123.75
	*d*_4_	50.71	45.47	0.0712	1.288	**119.53**
	*d*_5_	50.60	47.05	0.0741	1.325	121.77
	*d*_6_	51.56	46.60	0.0858	1.345	127.88
	*d*_7_	51.92	45.02	**0.0711**	1.311	121.38
	*d*_8_	**50.14**	44.17	0.0783	1.471	127.45
(1.06–1.1]	none	205.12	116.12	0.0968	1.752	298.71
	*d*_0_	62.40	**42.86**	0.1222	1.680	162.68
	*d*_1_	66.30	44.47	0.1015	1.234	143.26
	*d*_2_	68.65	47.14	0.1274	1.270	153.13
	*d*_3_	66.55	44.30	0.0924	**1.226**	140.68
	*d*_4_	63.14	43.29	**0.0917**	1.250	138.01
	*d*_5_	60.32	45.17	0.0924	1.288	**136.81**
	*d*_6_	59.55	44.45	0.1000	1.323	139.66
	*d*_7_	63.27	43.70	0.0962	1.284	140.79
	*d*_8_	**59.19**	42.88	0.0963	1.450	142.80
(1.1–1.14]	none	232.79	102.91	0.1262	1.726	336.04
	*d*_0_	71.69	38.74	0.1404	1.627	174.83
	*d*_1_	77.47	38.97	0.1196	**1.198**	157.01
	*d*_2_	76.18	44.26	0.1448	1.242	162.36
	*d*_3_	75.79	43.19	0.1256	**1.198**	156.50
	*d*_4_	75.80	**37.43**	**0.1125**	1.212	154.47
	*d*_5_	71.56	40.91	0.1170	1.246	**152.74**
	*d*_6_	**70.74**	40.48	0.1182	1.288	154.07
	*d*_7_	74.66	38.51	0.1157	1.245	155.50
	*d*_8_	70.83	37.96	0.1167	1.412	159.04
(1.14–1.18]	none	251.09	90.39	0.1528	1.696	360.18
	*d*_0_	85.37	**26.26**	0.1527	1.570	188.78
	*d*_1_	87.40	31.49	0.1428	1.155	168.80
	*d*_2_	83.02	39.99	0.1608	1.209	169.82
	*d*_3_	89.38	31.74	0.1438	**1.154**	170.87
	*d*_4_	85.46	31.63	0.1463	1.170	168.17
	*d*_5_	82.66	32.97	0.1397	1.201	165.97
	*d*_6_	**78.04**	35.72	**0.1391**	1.259	**164.15**
	*d*_7_	83.54	32.99	0.1408	1.223	168.11
	*d*_8_	84.23	26.82	0.1443	1.368	176.58

We can see that a great majority of bad (boxed) results is associated to no-AQM scheme. Moreover, in no single case the best result is associated with no-AQM scheme. These are very strong arguments for using AQM.

## 6 Conclusions

We presented the results of an experiment carried out in our university network, in which an AQM device, designed and programmed for this experiment, was running. In particular, the class of AQM algorithms, in which the packet dropping probability is a function of the queue size, was used in the device. Nine dropping function shapes, known from the networking literature, were implemented and tested, in addition to the classic FIFO queue. Several commonly used performance characteristics, including the load, queue size, loss ratio and burst ratio were recorded during a month of measurements. In addition, two cost functions, expressing the combined impact of these characteristics, were computed. What is important, the AQM device itself did not introduce any limitations on the network throughput—its internal performance was far better than needed to fully saturate the 1Gb/s link used. This was confirmed in laboratory stress tests.

From the obtained results, we can draw at least three clear conclusions.

Firstly, an application of the AQM with any of the non-trivial dropping functions, *d*_1_-*d*_7_, proved to be highly beneficial, if compared with no AQM. Typically, the queueing delay, the stability of the queue and the burst ratio ware improved by far, while the loss ratio was kept at the similar level, as in the no-AQM case.

Secondly, it was not crucial, which particular shape of the dropping function, taken from the literature, was used. All the shapes used in the measurements, except for the trivial *d*_0_ and *d*_8_, provided a very good performance, much better than the lack of AQM. Naturally, some of the non-trivial functions, *d*_1_-*d*_7_, proved to be slightly better than others (e.g. *d*_4_). The differences, however, are not critical—any one among *d*_1_-*d*_7_ can be recommended to replace the classic FIFO buffer.

Thirdly, the study underlined the necessity to perform experiments and measurements of this type in real, operating networks. The very complex nature of real traffic, with distributed round trip times among flows, large number of distinct protocols, different flow characteristics and durations, lead sometimes to different conclusions, than those obtained via simulation models. For instance, the lowest loss ratio in the whole range of load was observed here for one of the dropping functions, rather than for the FIFO buffer, as predicted by simulations, [36]. Also, the best results in simulations were obtained for a convex dropping function, which was speculated to be a consequence of some special properties of convex functions (e.g. [37]). This effect was not confirmed herein. In fact, the convex dropping function *d*_2_ performed slightly worse than other non-trivial functions.

It is hard to explain the detailed impact of the shape of a particular dropping function on the system performance. This is again due to the nature of real traffic, which is not only very complex at any particular moment in time, but also highly variable. The overall load may vary by far, proportions between TCP and UDP traffic may vary by far, the number of flows and data type they carry may vary by far, statistical properties of flows (rates, packets sizes, round trip times, durations) may vary by far, etc. Each of the mentioned traffic features influences the resulting performance characteristics, when combined with a particular shape of the dropping function. Therefore, the only reasonable way to study this is by exposing each of the tested dropping function to different traffic types, many times, and in a long period of time. This was done in the experiment described in the paper.

There are several possible directions of future work. Firstly, other networks can be used to carry out similar experiments. Before that, we cannot be sure, to what extend the results obtained in one network, are representative to other networking environments. Secondly, a much more detailed analysis can be performed. For instance, in our study we collected and discussed the performance characteristics for the aggregated traffic only. There was no distinction between individual flows in the measurements, thus the results are averaged across all the flows. It would be interesting to see, how different dropping functions influence the characteristics of individual flows (the per-flow loss ratio, the per-flow burst ratio etc), depending on the flow rate, its round trip time, and so on. Thirdly, AQM algorithms of different types than herein can be implemented and tested in real networks. Such study is needed not only to test the advantages and disadvantages of a particular algorithm in a real networking environment, but also to demonstrate the possibility of its implementation, efficient enough to make the algorithm useful in contemporary networks.
